# Genomic Selection for Fruit Quality Traits in Apple (*Malus×domestica* Borkh.)

**DOI:** 10.1371/journal.pone.0036674

**Published:** 2012-05-04

**Authors:** Satish Kumar, David Chagné, Marco C. A. M. Bink, Richard K. Volz, Claire Whitworth, Charmaine Carlisle

**Affiliations:** 1 The New Zealand Institute for Plant & Food Research Limited, Havelock North, New Zealand; 2 The New Zealand Institute for Plant & Food Research Limited, Palmerston North, New Zealand; 3 Biometris, Wageningen University and Research Centre, Wageningen, The Netherlands; Nanjing Agricultural University, China

## Abstract

The genome sequence of apple (*Malus×domestica* Borkh.) was published more than a year ago, which helped develop an 8K SNP chip to assist in implementing genomic selection (GS). In apple breeding programmes, GS can be used to obtain genomic breeding values (GEBV) for choosing next-generation parents or selections for further testing as potential commercial cultivars at a very early stage. Thus GS has the potential to accelerate breeding efficiency significantly because of decreased generation interval or increased selection intensity. We evaluated the accuracy of GS in a population of 1120 seedlings generated from a factorial mating design of four females and two male parents. All seedlings were genotyped using an Illumina Infinium chip comprising 8,000 single nucleotide polymorphisms (SNPs), and were phenotyped for various fruit quality traits. Random-regression best liner unbiased prediction (RR-BLUP) and the Bayesian LASSO method were used to obtain GEBV, and compared using a cross-validation approach for their accuracy to predict unobserved BLUP-BV. Accuracies were very similar for both methods, varying from 0.70 to 0.90 for various fruit quality traits. The selection response per unit time using GS compared with the traditional BLUP-based selection were very high (>100%) especially for low-heritability traits. Genome-wide average estimated linkage disequilibrium (LD) between adjacent SNPs was 0.32, with a relatively slow decay of LD in the long range (*r* ^2^ = 0.33 and 0.19 at 100 kb and 1,000 kb respectively), contributing to the higher accuracy of GS. Distribution of estimated SNP effects revealed involvement of large effect genes with likely pleiotropic effects. These results demonstrated that genomic selection is a credible alternative to conventional selection for fruit quality traits.

## Introduction

During the last 10 years, genome sequences of about 20 plant species including some from the Rosaceae family were made publicly available [1]. In 2010, an international consortium published the first draft of the apple (*Malus×domestica* Borkh.) genome sequence using DNA from a popular apple variety ‘Golden Delicious’ [2]. The apple genome sequence provided insight into the evolution of this globally important fruit crop, and is now being used to speed up the development of new varieties. Availability of genome sequence information along with high throughput genotyping platforms is changing the nature of research experiments to understand evolution of organisms, as well as transforming the strategies for genetic improvement. One such artificial selection strategy, called genomic selection (GS), is revolutionizing the genetic improvement of animals and plants species [3]–[5].

Standard apple cultivar breeding follows three stages. The first stage (stage-1) is to identify parents from the pool of available candidates, cross them and select the best offspring from large families. In stage-2, multiple copies of the selections are propagated onto clonal rootstock for trial across different environments, while in stage-3, larger-scale testing of the best selections is conducted, often on commercial orchards. Phenotypic data from stage-1 can be analysed using individual-tree mixed models to obtain best liner unbiased prediction (BLUP) of breeding values (BVs) of seedlings. Various apple breeding programmes now routinely use BLUP-BVs for making selections (e.g. [6]). It generally takes about 7 years from seed before outstanding individuals can be identified for further use as a parent or potential stage-2 cultivar. This leads to a long generation interval, substantial costs and complex logistics for phenotypic recording. Comparatively, BV estimated with genome-wide distributed markers (GEBV) is likely to increase annual genetic gain because of a reduced generation interval, and thus genomic selection (GS) is now being implemented in various animals and plants species [3], [7]–[9]. Calculation of GEBV requires a population with information on genetic markers and phenotypes, called the ‘training’ population. BLUP-BV of training individuals are first used to estimate effects for the genetic markers, which can then be used to calculate GEBV of individuals with only marker information, called the ‘selection’ population. The accuracy of GEBV will depend on the number of observations, heritability of the trait, the number of markers and the linkage disequilibrium (LD) among these markers [10]–[12].

Methods for deriving GEBV differ in terms of the prior assumptions about the distribution of the effects of the single nucleotide polymorphisms (SNPs). Ridge-regression BLUP (RR-BLUP), where the effect of each marker is assumed to come from a normal distribution with equal variance across all markers, is simple to understand and implement [3]. In RR-BLUP, estimates of marker effects are penalized to the same extent, and this may not be appropriate if some markers are located in regions not associated with genetic variance, whereas others are linked to quantitative trait loci (QTL) [13]. To overcome this limitation, Bayesian methods using marker-specific shrinkage of effects, such as BayesA and BayesB, have been proposed [3]. A popular alternative is the Bayesian Least Absolute Shrinkage and Selection Operator (LASSO) [14], which allows for departures of SNP effects from normality (i.e., some SNPs of big effect but a large proportion of SNPs have close-to-zero effect) while still allowing for shrinkage (e.g. [15]).

DNA markers located close to major causal loci controlling disease resistance have been used for selection by apple breeders in the last 10 years [16]. However, for more complex traits that are controlled by several loci such as many aspects of apple fruit quality (firmness, astringency, soluble solids, acidity, etc.), breeders have used estimated breeding values (EBV) of individuals, based on their phenotype. Since any one locus captures only a small portion of the total genetic variance for complex traits, a large number of genome-wide markers are required for making accurate selection decisions. Publication of the first draft of the apple genome was followed by re-sequencing of 27 apple cultivars that are founders in global apple breeding programmes. These efforts produced a huge reservoir of DNA markers, which led to the development of the first apple Infinium SNP chip comprising nearly 8,000 markers [17]. This SNP assay is a crucial tool for the application of GS for complex traits. In this study, we used this SNP assay for the first time, and compared RR-BLUP and Bayesian LASSO for their accuracy of predicting GEBV for fruit quality traits in a training population of 1,200 individuals. We then used the observed distributions of SNP effects as a mechanism for understanding differences in the genetic architecture of traits, and to identify genomic regions with probable pleiotropic effects. We also investigated the size and the decay of LD in our breeding population. To our knowledge, this is the first study of evaluating GS for any cross-pollinating fruit crop species.

## Results

### SNP genotyping, SNP density, and linkage disequilibrium

Problematic seedlings that had many missing data were discarded, resulting in a training set of 1,120 individuals. Out of 7692 SNPs on the IRSC apple Infinium array v1, 20% had *GenCall* score lower than 0.15, 10% were monomorphic, and a further 30% were discarded due to thresholds used for *ClusterSep* and *50%GC* score. After further screening for frequency of missing calls, allele frequencies, and segregation discrepancies, a high quality set of 2,500 SNPs with an average call rate of 98% was retained for developing GS prediction models. The number of SNPs dropped varied from 58% to 70% for various linkage groups (LG) (Table 1). The retained 2,500 SNPs were evenly spread across the apple genome, i.e. the proportion (out of 2500) of SNPs on any given LG was generally similar to the relative size of that LG assuming the total genome size as 1300 cM (Table 1). The average distance between all adjacent marker pairs was 0.240 megabase (Mb), while the maximum distance between adjacent SNPs varied from 1.523 Mb on LG4 to 6.206 Mb on LG15 (Table 1).

**Table 1:** Relative size of linkage groups (LG) (assuming the genome size of 1300 cM), and the number of single nucleotide polymorphisms (SNPs) retained on each LG after various quality checks.

LG	Relative size (%)	Initial No. of SNPs	No. of SNPs retained	Average distance (megabase)	Maximum distance (megabase)
1	6.62	434	143	0.252	2.599
2	6.31	684	243	0.161	3.534
3	6.58	487	148	0.267	3.055
4	4.84	386	139	0.177	1.523
5	6.79	486	162	0.232	4.352
6	5.61	340	107	0.282	5.362
7	4.37	340	120	0.255	1.873
8	5.43	399	129	0.272	3.446
9	5.41	477	202	0.177	2.607
10	6.99	531	136	0.272	3.073
11	5.74	456	155	0.257	3.306
12	5.48	459	147	0.245	1.925
13	5.72	423	127	0.315	2.543
14	5.69	374	113	0.296	3.945
15	8.60	621	200	0.273	6.206
16	4.31	347	119	0.188	3.090
17	5.56	448	110	0.245	1.492

The average distance and the maximum distance between adjacent SNP pairs are also shown for each LG.

**Figure 1:**
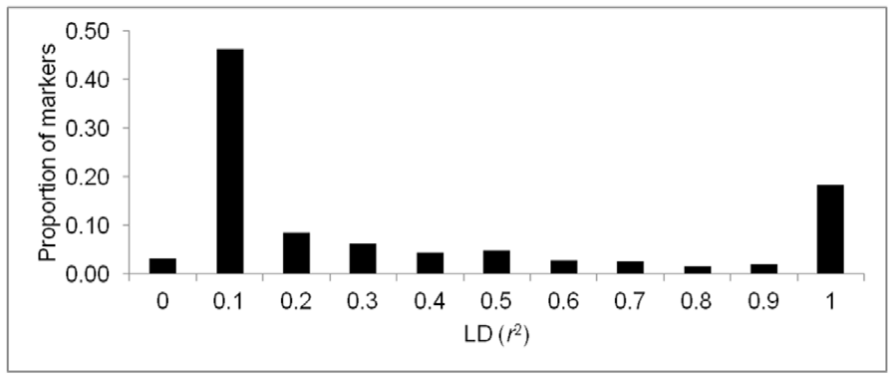
Distribution of linkage disequilibrium (LD), measured with *r* ^2^, among adjacent single nucleotide polymorphisms (SNPs) pairs in the training population.

**Figure 2:**
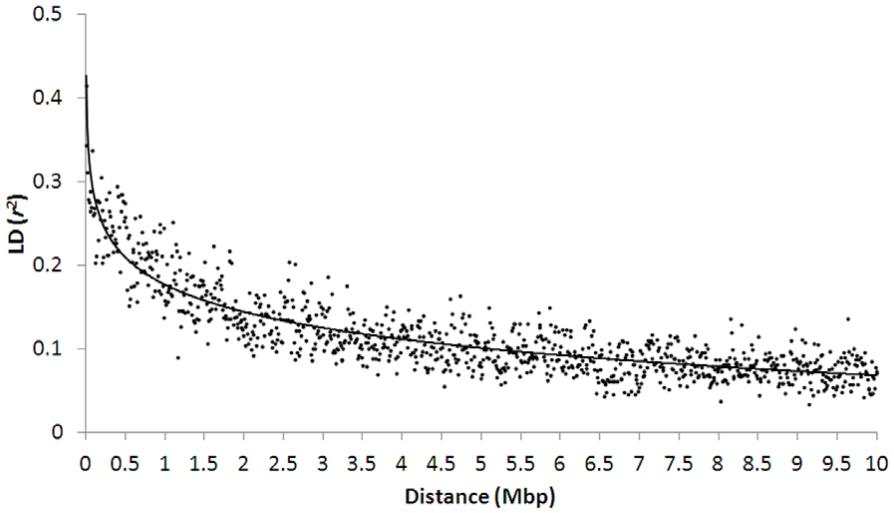
Average linkage disequilibrium (LD) measured as *r* ^2^, for pairs of single nucleotide polymorphisms (SNPs) in increments of 10,000 bp, according to the distance between SNPs.

The average LD (*r* ^2^) between adjacent SNPs pairs in the training population was 0.32. Three percent of the adjacent marker pairs had *r* ^2^ = 0 and 17% had *r* ^2^ values ranging between 0.90 and 1.00 (Figure 1). To understand the pattern of LD decay, estimates of pair-wise LD were averaged in the increments of 10 kilobase (kb) distance between SNPs. A high degree of LD was observed even at longer distances between markers; for example, the average *r* ^2^ for SNPs separated by 100 kb, 500 kb (which approximately equates to 1 cM in apple), and 1000 kb was 0.33, 0.25, and 0.19, respectively (Figure 2).

### Accuracy of genomic selection models

Accuracy of predicting unobserved BLUP-BVs was almost identical for the RR-BLUP and Bayesian LASSO methods, and varied from about 0.68 (for astringency (AST)) to 0.89 (for soluble solids (SSC)), whereas for all other traits, the prediction accuracies were similar and ranged between 0.81 and 0.83 (Table 2). The standard errors of prediction accuracies, calculated from the validation replications, were 0.01 or 0.02 for various traits. The difference between the minimum and the maximum (across 10 replicated sets) accuracy varied from 0.07 (for SSC) to about 0.12 (for fruit firmness (FF)), consistent for both methods, suggesting some variation between replicated sets (results not tabulated). The average regression coefficient for both methods was close to one, with RR-BLUP showing slightly larger bias than Bayesian LASSO (Table 2). The degree of bias for titratable acid (TA) was high, and similar for both methods. Estimated standard errors of regression coefficients were slightly larger than those for correlation coefficients (Table 2).

**Table 2:** Average predicted accuracy (correlation) and bias (regression) of Bayesian LASSO (BL) and RR-BLUP methods for various traits: fruit firmness (FF), soluble solids (SSC), russet, weighted cortex intensity (WCI), astringency (AST), titratable acidity (TA).

	BL		RR-BLUP	
Trait	Correlation	Regression	Correlation	Regression
FF	0.83 (0.02)	1.01 (0.04)	0.83 (0.02)	1.04 (0.04)
SSC	0.89 (0.01)	1.01 (0.02)	0.89 (0.01)	1.02 (0.02)
Russet	0.81 (0.02)	1.00 (0.03)	0.82 (0.02)	1.02 (0.03)
WCI	0.83 (0.02)	1.01 (0.03)	0.82 (0.02)	1.04 (0.03)
AST	0.68 (0.01)	1.00 (0.05)	0.67 (0.01)	1.03 (0.06)
TA	0.81 (0.02)	1.09 (0.05)	0.81 (0.02)	1.09 (0.05)

Standard errors are shown in parentheses.

Accuracy of one of the GS models (RR-BLUP) was also compared with the conventional BLUP-based selection (Table 3). Estimates of *h* ^2^ varied from 0.16 (for TA) to 0.60 (for russet), and contributed to the observed accuracies of conventional- and genomic selection methods for various traits. The accuracy of BLUP-based selection varied from 0.73 to 0.84 for various traits, while accuracies of GEBV-based selection were >0.90. Some of the estimated correlations between GEBV and TBV were outside the parameter space (>1.0), so these were constrained to a maximum theoretical value of 1.0. The selection response per year was higher for GS for all traits considered, with the efficiency of GS being 100% to 141% higher than that of BLUP-based conventional selection for various traits (Table 3).

**Table 3:** Relative efficiency of GEBV-based selection compared with the conventional BLUP-based selection for various traits: fruit firmness (FF), soluble solids (SSC), russet, weighted cortex intensity (WCI), astringency (AST), titratable acidity (TA).

Trait	*h* ^2^	*r*(BLUP-BV, TBV)	*r*(GEBV, TBV)	Efficiency	Increase (%)
FF	0.43	0.79	1.0*	2.21	121
SSC	0.19	0.73	1.0*	2.39	139
Russet	0.60	0.84	0.96	2.00	100
WCI	0.26	0.75	1.0*	2.34	134
AST	0.26	0.75	0.90	2.12	112
TA	0.16	0.73	1.0*	2.41	141

Estimates of narrow-sense heritability (*h* ^2^) are also shown for each trait.

* Estimated correlation was outside parameter space (>1.0), so constrained to 1.0.

### Distribution of SNP effects

**Figure 3:**
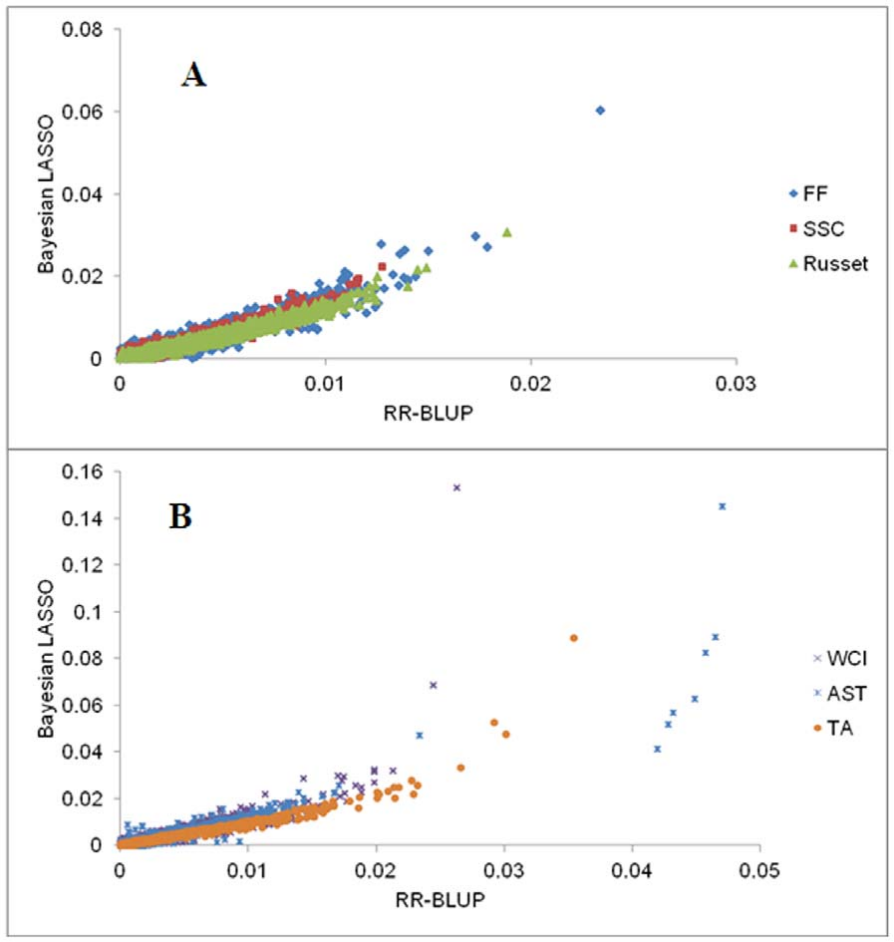
Relationship between single nucleotide polymorphisms (SNPs) effects obtained from RR-BLUP and Bayesian LASSO for various traits. A: Fruit firmness (FF), soluble solids (SSC), and Russet; B: Weighted cortex intensity (WCI), astringency (AST), and titratable acidity (TA).

**Figure 4:**
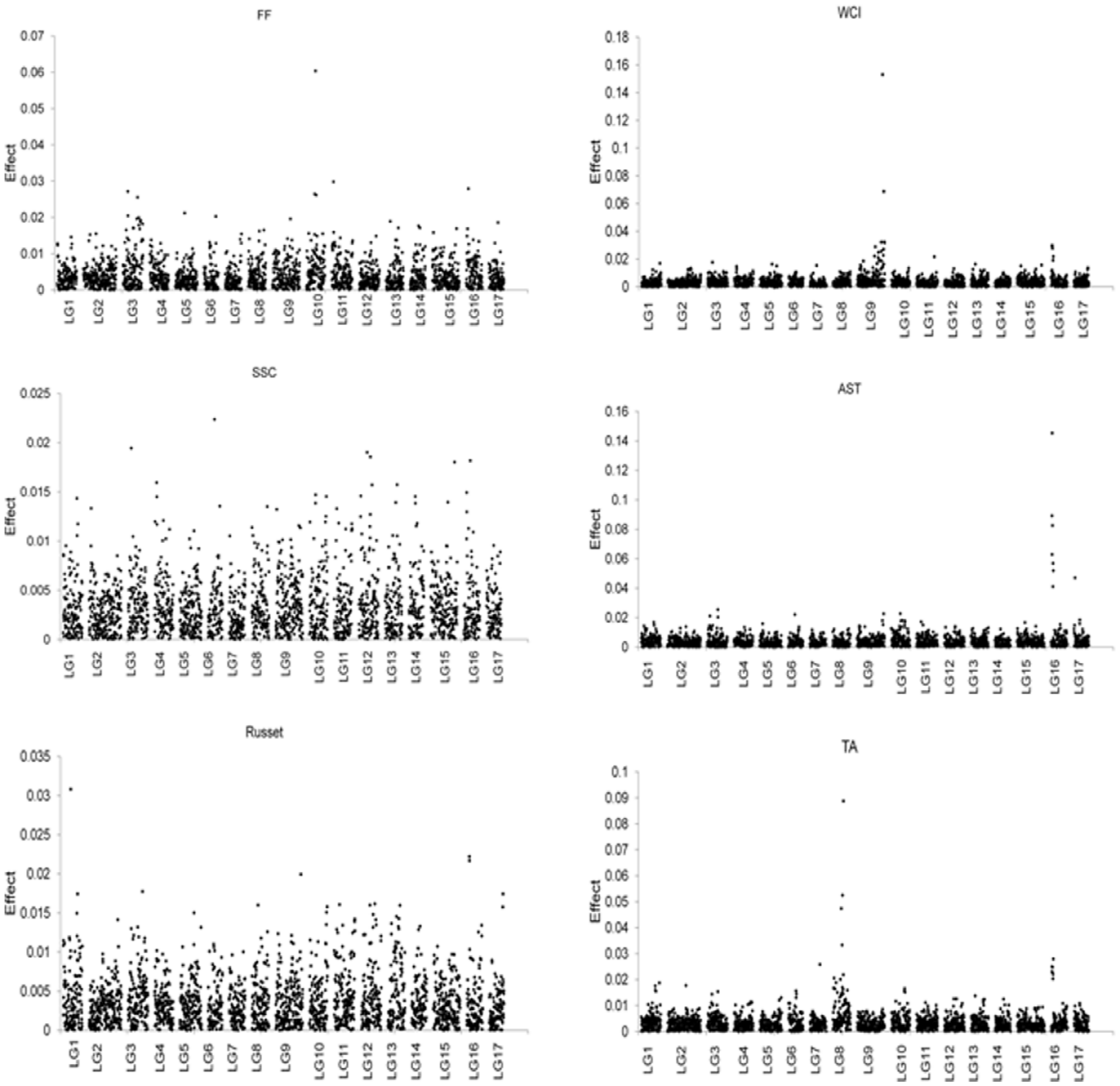
Estimates of SNP effects (in additive genetic standard deviation) obtained using Bayesian LASSO for various traits: Fruit firmness (FF); Soluble solids (SSC); Russet; Weighted cortex intensity (WCI); Astringency (AST); Titratable acidity (TA). Effects are shown for each linkage group (LG: 1 to 17) across the genome.

Ranking of SNPs in terms of the size of their individual effect on a trait was very similar for RR-BLUP and Bayesian LASSO, except that the latter induced stronger shrinkage of estimates for SNPs with relatively small effect and less shrinkage of estimates for SNPs with sizable effect (Figure 3A, B). Estimated correlation between SNP effects from the two methods was >0.92 for all traits except for WCI (0.81), and the three SNPs with the largest effects for any given trait were common to both methods. Thus, we have only presented here the genome-wide distribution of SNP effects estimated using the Bayesian LASSO (Figure 4).

The SNP with the largest (more than 10 times the average) effect on FF was located on LG10, and this SNP is a T/G variant located within the first exon of the *polygalacturonase* (*PG*) gene (MDP0000232611), 20.833 kb from the top of LG10. In addition, there were few SNPs with moderate effect on FF located on LGs 3, 11 and 16 (Figure 4). The five SNPs with highest effects on SSC were located on LGs 3, 6, 12, 15 and 16, with the effect of the top one on LG6 being about six time higher than the average effect (Figure 4). The SNP marker with the largest effect on russet coverage was located on LG1, while genomic regions of moderate effect were also identified on LGs 9, 16 and 17 (Figure 4). A SNP with a massive effect (more than 40 times the average effect) on WCI was located on LG9 (Figure 4). This SNP marker on LG9 is a T/C variant and is located within the second exon of the *MdMYB10* gene (MDP0000259616), 32.840 kb from the bottom of LG9. A chromosome segment on LG16 also appeared to have some influence on the expression of WCI (Figure 4). A cluster of SNPs with a very large effect on AST was located on LG16, and an additional SNP located on LG17 appeared to have a moderate effect on AST variation (Figure 4). The SNP with the largest effect (about 25 times the average) on TA was located on LG8, while a cluster of SNPs with a moderate effect was located on LG16 (Figure 4). The same cluster of SNPs on LG16 had large effects on WCI, AST and TA and spanned the *Leucoanthocyanidin Reductase* (*LAR1*) gene (MDP0000376284) that is located between 1.496 kb and 1.669 kb on the top of LG16. A summary of the largest SNP effects for each trait is presented in Table 4.

**Table 4:** SNPs with the largest effects (in additive genetic standard deviation) on fruit firmness (FF), soluble solids (SSC), russet, weighted cortex intensity (WCI), astringency (AST) and titratable acid (TA).

Trait	SNP (NCBI db)	Linkage group & position (bp)	Effect	Heterozygosity	Gene name & ID
FF	ss475883584	LG10 (20,833,228)	0.06	0.50	*Polygalacturonase* (*PG*); MDP0000232611
SSC	ss475878574	LG6 (12,001,079)	0.02	0.42	Unknown
Russet	ss475876799	LG1 (18,714,053)	0.03	0.42	*40S ribosomal protein;* MDP0000284030
WCI	ss475879555	LG9 (32,840,325)	0.16	0.18	*MdMYB10;* MDP0000259616
AST	ss475881697	LG16 (1,540,624)	0.15	0.40	*Leucoanthocyanidin Reductase* (*LAR1*); MDP0000376284
TA	ss475882883	LG8 (19,658,610)	0.09	0.43	*RING finger and CHY zinc finger domain-containing protein;* MDP0000294924

## Discussion

### Efficiency of genomic selection

The BLUP-BVs obtained from equation 1 were used as such for developing GS models, but some previous studies (reviewed by [18]) have used de-regressed BLUP-BVs instead. Recent studies (e.g. [19]–[20]) have shown that using BLUP-BVs, as opposed to de-regressed BVs, as phenotypes for genomic predictions, resulted in higher accuracies of GS – supporting the approach used in our study. Results showed that unobserved BLUP-BVs for a range of commonly assessed fruit quality traits can be predicted with average accuracy of about 0.80.

It was demonstrated by [21] that to predict GEBV with an accuracy of about 0.90, 10*N*e*L* markers are required, where *N*e is the effective population size and *L* is the length of the genome in Morgans. Most apple breeding programmes worldwide are based on narrow genetic pools. Using historical pedigree records of commercial apple cultivars, [22] estimated the ‘status number’ (which is a measure of *N*e that is based on current relatedness only; see [23]) of the top-50 mainstream cultivars to be 8. Since apple breeding populations are developed using commercial cultivars as well as advanced selections, it is likely that the effective population size of such populations would be higher, which would require higher marker density . The observed average accuracy of 0.80 suggests that a significant amount of genetic variation, likely due to low-frequency alleles, could not be captured with the SNP density and the training population size used in this study. Efforts are underway to increase both the density of SNP arrays and the number of training individuals, in order to achieve higher accuracy (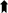 0.90) of GS for the key selection traits.

In addition to the number of markers used, the accuracy of GS is also dependent on the LD between markers and QTLs. The SNP markers are required to be in sufficient LD with the QTL so that GS is effective especially across generations. However, the QTL cannot be observed directly, and thus LD between SNPs can be used as a surrogate to evaluate the extent of LD in the population of interest (e.g. [24]). The combination of long-distance LD due to pedigree relatedness (e.g. full sibs and half sibs) and short-distance ancestral LD due to small effective population size are among the key features of our training population, resulting in the high observed LD (mean *r* ^2^ = 0.32). However, the LD decay can vary significantly between different types of genetic materials, so efforts are now being made to better understand LD patterns in apple germplasm, breeding populations and commercial cultivars. As long as the mean *r* ^2^ between adjacent SNPs was >0.2, GEBV could be predicted accurately at least in the one or two successive generations before needing recalibrating of SNP effects [3], [25]. Approximately 10% of the adjacent SNP pairs had a physical distance >1,000 kb, and the average observed *r* ^2^ of <0.2 between such markers (Figure 2) would suggest that higher SNP density than currently used could further improve GS accuracy.

When the training data for GS consist of individuals from reproductively isolated ancestral populations, estimates of marker effects may be biased due to population stratification and admixture [26]. However, this probably was not an important issue in our work. The six parents used were advanced selections (or cultivars) sampled from the same breeding population, and therefore the issue of population structure should not be critical. Also, the known genetic relationships of the training individuals were taken into account *via* additive genetic relationship matrix to derive BLUP-EBVs, which were used as ‘phenotype’ for developing GS models. Moreover, provided high-density SNPs are used and analyzed simultaneously, as in this study, admixed populations can be used to develop reliable GS prediction equations even if pedigree and breed (or population) origin has not been explicitly modeled to avoid spurious signals [26].

We aim to apply the model derived from the training population to a selection population consisting of 2,000 young seedlings generated from 10 full-sib families. The pollen parents of these 10 families were selected from our training population and the seed parents (some of which have genetic relatedness with some of the six parents of our training population) were identified from previous progeny trials, so providing a strong genetic link between training and selection candidates, which will also provide higher GEBV accuracy [10]. All 2000 seedlings in the selection population will be genotyped for the same SNP markers used in the training population, in order to obtain their GEBV for each trait. Using an index of GEBV, the top-ranked (say, 20) seedlings will be identified and then multiple copies of these selections will be propagated onto clonal rootstock for stage-2 trials. The remaining seedlings will be transferred to a nursery for later planting in the orchard. Once phenotypic data from the selection population becomes available in the year 2016, we will compare their observed BLUP-BV with GEBV and also compare the predicted and observed genetic gain.

When a training population is established in one environment but the aim is to select individuals for multiple environments, the realized accuracy of GS could be lower at the sites that are not represented in the training set [27]. The reduction in accuracy would depend on the magnitude of genotype-by-environment interaction (G×E) for selection traits. There is not enough information available on the effect of G×E on fruit quality traits at the clonal level, but such effects were found to be minimal at the family level, across the key apple growing areas in New Zealand [6].

Estimated correlation between GEBV and TBV were higher than those between BLUP-BV and TBV (Table 3). RR-BLUP method used in this study is essentially similar to a BLUP model that uses marker-based (say, realized) genetic relationship matrix, and the use the realized relationship matrix instead of the average relationship matrix has been shown to substantially increase the accuracy of breeding values [28]. Thus results from our empirical study are in agreement with those based on simulation studies (e.g. [28]). Relative efficiency of GS compared to the conventional BLUP selection was higher for traits with low heritability; for example, efficiency of GS was 100% and 141% higher than BLUP-based selection for russet (*h* ^2^ = 0.60) and TA (*h* ^2^ = 0.16) respectively; supporting results from earlier studies (e.g. [29]).

### Distribution of QTL effects

Estimated SNP effects capture at least partly the underlying QTL effects, especially when LD estimates between adjacent markers are reasonable. Consequently, the distribution of estimated SNP effects should resemble the distribution of the underlying QTL effects. The SNP array used in this study was designed to encompass SNPs in the coding region of predicted gene models and some candidate genes such as, *MdMYB10*, *MdPG*, and *MdLAR* [17]. The SNP showing the largest effect on FF on LG10 (Figure 4) reside in the polygalacturonase (*PG*) ethylene-related gene, which depolymerizes cell wall pectin and the involvement of this gene in fruit softening process was recently confirmed in a cross between ‘Fuji’ and ‘Mondial Gala’ [30]. The second and the third largest SNP effects were located on LGs 11 and 16 respectively, but there are no known candidate genes at these genomic positions. However, an earlier study using a bi-parental population reported a moderate size QTL on LG11 [31]. No genomic region of very large effect on SSC was observed in our study (Figure 4), which is consistent with previous reports [31]–[32]. For russet, one QTL of large effect was identified on LG1, and a couple with moderate effects located on LGs 9 and 16, suggesting a polygenic control of russet and SSC. Our unpublished results showed moderate genetic correlation between SSC and russet, which would suggest some common genes with effects on both traits. In fact, out of the 12 largest effect SNPs for both SSC and russet, four were common to both traits, suggesting some pleiotropic effects.

Red colour in apple flesh results from high concentration of anthocyanins. Seedlings of two red flesh phenotypes, putatively named Type 1 and Type 2 [33], were present in our training population. Type 1 red flesh is characterized by red pigmentation throughout the fruit core, cortex, and foliage; and Type 2 is characterized by red pigmentation in the fruit cortex only, with white fruit core and green foliage. The role of *MdMYB10 gene* on anthocyanin biosynthesis in Type 1 red flesh apple was demonstrated using transient approach in tobacco, stable transformation in apple, and by mRNA transcript profiling in red flesh apple fruit [34], and this gene has been mapped to LG9 [35]. However, *MdMYB10* marker is unlinked to Type 2 red flesh phenotype [33], and perhaps there are numerous low-frequency and small-effect loci contributing to the expression of Type 2 phenotype. The SNP marker associated with weighted cortex index (WCI) in our experiment is located in the second exon of *MdMYB10*, which is physically close to the R6 motif [34]. WCI trait was derived (see Methods section) from two separate phenotypes (i.e. the intensity of red colour, and the proportion of cortex with red colour), both of which vary between seedlings of each red flesh types; suggesting involvement of various small effect genes and a large influence of environment. All these factors would have contributed to the low observed *h* ^2^ (0.26) of WCI in this study.

A cluster of SNPs at the top of LG16 is associated with AST and WCI. This cluster of SNPs resides in the *MdLAR1* candidate gene. *LAR1* is a key enzyme in the flavonoid biosynthetic pathway, reducing leucoanthocyanidin into the flavanol compound catechin, a monomer of condensed tannins (also known as proanthocyanidins). This reaction branches off from the cyanidin biosynthetic pathway. It is likely that condensed tannins (CTs) act as a co-pigment of cyanidin to create more intense red coloration in the fruit and hence the effect on WCI. Furthermore, CTs are known for their role in imparting astringency to fresh fruits, juices and wine. *MdLAR1* was linked to QTLs controlling fruit skin and cortex concentrations of CTs in a ‘Royal Gala’×‘Braeburn’ population [36].

Distribution of SNP effects for TA (Figure 4) suggested that one major QTL on LG8 and a moderate effect QTL on LG16 exist in our population, supporting earlier results from bi-parental QTL mapping studies [31]–[32]. The distribution of TA phenotype in our training population was normal, suggesting that numerous small effect genes contributed to its expression despite one or two large QTLs identified. The six parents of the training population do not represent the TA variation available in wider breeding material, hence contributing to the low observed *h* ^2^ (0.16) compared to some other studies (e.g. [37]). To our knowledge there are no published reports of known candidate genes for TA on LG8. A SNP with a moderate effect on TA (Figure 4) is located close to the malic acid gene (*Ma*), which was mapped to LG16 [38]. A gene (*Mal-DDNA*), isolated by [39], was shown to be expressed differentially in low- and high acid genotypes, but it appears that this gene has not yet been genetically mapped. Interestingly, it is the same cluster of six SNPs on LG16 that is associated with WCI, AST and TA, suggesting a possible pleiotropic effect of this genomic region.

### Comparison of prediction models

The RR-BLUP and Bayesian method provided similar accuracy of GEBV (Table 2), and there are some empirical studies in animal breeding supporting these results (e.g. [11]–[12], [19]). Distribution of QTL effects, as inferred from estimated SNP effects, varied considerably between traits (Figure 4). Despite these contrasting distributions, RR-BLUP and Bayesian LASSO performed very similarly on these traits. Using data from a German Holstein cattle population, [40] also reported similar accuracies of RR-BLUP and BayesB models for milk-fat yield, which is controlled by a major gene *DGAT1*. Interestingly the highest accuracy of GS was achieved for SSC which is characterized largely by QTLs of small effects. Perhaps in the presence of few QTLs of large effects, it becomes essential to have SNPs in high LD with these large effects in order to obtain higher accuracy of GS. Even in the presence of large effect QTLs, there might be a large number of small loci for which no clear evidence is found for a direct association, but together these loci still may explain a substantial part of the BV and thus make different GS models less separable. Bayesian shrinkage regression methods primarily capture LD, while the RR-BLUP method captures genetic relationships and polygenic resemblance [10], [41]. Somewhat higher LD, and high genetic relationships between training and validation sets in our study could have played in favour of both methods and thus similarly high prediction accuracy was observed for RR-BLUP and Bayesian LASSO.

### Introgression of new traits

Introgression of monogenic disease resistances from an inferior donor into high-quality recipient apple cultivars is generally performed by backcrossing. Although GS can be used to achieve this goal, the introgression of favourable alleles from the donor parent cannot be guaranteed unless the SNP density is such that at least one SNP allele is in high LD with the functional mutation. Introgression of monogenic traits could be fast-forwarded using a two-step approach, i.e. gene-assisted selection (GAS) for monogenic traits followed by GS for oligogenic or polygenic traits. This strategy is currently being implemented in our apple breeding programme.

### Conclusion

Distribution of estimated SNP effects suggested genes of major effect especially for traits such as WCI, TA, and AST, while large effect QTLs appeared to be involved in the expression of FF and russet. Various factors including training population size, number of SNPs, and magnitude of LD led to similar accuracies of the RR-BLUP and Bayesian LASSO methods. Thus, either of these two models could be used in practical applications of GS in apple breeding programmes. Relative gain per unit time from GS compared with the traditional BLUP-based selection were very high (>100%) especially for low-heritability traits. Because of the high degree of genetic relatedness among the commonly used parents, the model developed in this study could be applicable to other apple cultivar breeding populations. Based on the high accuracy of GS in our study, we conclude that if the objective of any apple breeding programme is to accelerate the breeding cycle by making selections prior to extensive fruit-quality phenotyping, GS shows strong potential as a means of achieving this goal.

## Materials and Methods

### Training population and fruit assessment

A set of four white-fleshed female parents (NZSelectionT153, NZSelectionT179, ‘Sciros’ and ‘Fuji’) and two red-fleshed pollen parents (NZSelectionT31 and NZSelectionT51) were crossed in a factorial (4×2) mating design, except that the cross between NZSelectionT179 and NZSelectionT31 was unsuccessful leaving seven full-sib families. The pollen parents NZSelectionT31 and NZSelectionT51 represent Type 1 and Type 2 red flesh phenotype, respectively [33]. Seedlings numbers varied between families, ranging from 40 to 350, with a total population size of 1200. Seedlings were planted in a nursery in November 2005. Two-year-old seedlings were propagated onto ‘M. 9’ rootstock in 2007 and then in the following year planted into the orchard (Havelock North, New Zealand) at 3.0×0.5 m spacing for fruit evaluation. All trees received standard commercial management for nutrition, pesticide, fruit hand-thinning, and irrigation. No specific permission from the New Zealand regulatory authorities was required for this study. The location of this study is not protected in any way, and the study did not involve endangered or protected species.

Harvesting and fruit assessment began in the second season (February–May 2010) after orchard planting, and was repeated for a second consecutive year. Fruiting trees were harvested twice at 7- to 10-days intervals beginning when fruit were judged mature, based on a change in skin background colour from green to yellow, and when the starch pattern index was between 3 and 4. Samples of six fruit per harvest were stored for 70 days at 0.5°C, then a further 7 days at 20°C and evaluated. Six traits were evaluated on the fruit samples using instrumental, sensory, or visual assessment methods. Fruit flesh firmness (FF) was determined on opposite sides of each fruit after peel removal using a Fruit Texture Analyzer (GÜSS) fitted with an 11-mm diameter probe tip. Soluble solids concentration (SSC) for each fruit was measured with the juice from the probe using a digital refractometer (Atago PR-32). Average russet coverage (russet) and flesh astringency (AST) were scored for each sample on a scale from 0 ( = none) to 9 ( = highest) by two trained assessors. Fruit from each seedling were then cut in half across the equator and the proportion of the cortex area that was red (PRA) and the intensity of the red (RI) ( = 0 (none) to 9 (highest)) were scored. A weighted cortical intensity (WCI) was then calculated (PRA×RI) as an estimation of the amount of red pigment in the fruit. A cortical wedge (10 g) was then removed immediately from each half of each apple, combined for each seedling, juiced in a blender (Magimex Le Duo), and frozen. Titratable acidity (TA) was measured on the thawed juice using an automatic acid titrator (Metrohm 716 DMS) and the percentage of malic acid in fruit juice was recorded.

### Phenotypic data analysis

Individual fruit measurements (FF, SS, and WCI) were first averaged for each seedling, and then averaged over the two harvests in a given year. As repeated records for each seedling occurred over two years, there was an element of ‘permanent environmental effect’ associated with a seedling's performance. In other words, when a seedling has multi-year records, its breeding value and part of the environmental effects are repeated. We used the following individual-tree mixed linear model accounting for repeated records for each trait:
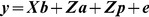
(1)where ***y*** is the vector of observations, ***b*** is the vector of fixed effects (e.g. assessor, year), ***a*** is a vector of additive genetic effects of seedlings, ***p*** is a vector of permanent environmental effects and ***e*** is a vector of residual effects. The matrix ***X*** is the incidence matrix for the fixed effects and ***Z*** is the incidence matrix relating observations to seedlings. Each seedling has an additive genetic as well as a permanent environmental effect, so both effects have the same design matrix (***Z***). The associated variances with the random effects, ***a***, ***p*** and ***e*** were 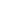, 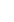 and 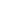 respectively. We assumed that within a seedling there was no correlation between its additive and its permanent environmental effect, and that permanent environmental effects for different seedlings are uncorrelated. Estimates of narrow-sense heritability (*h* ^2^) of each trait were obtained as the ratio of additive variance (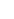) to the total phenotypic variance ( = 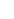
+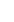
+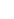). Genetic relationships among seedlings and among parents (some parents were related) were taken into account via the additive genetic relationship matrix. ASReml software [42] was used to obtain BLUP-BVs of all seedlings, which were later used for developing GS models.

### Single nucleotide polymorphism genotyping

The training population was genotyped using the International RosBREED SNP Consortium (IRSC) apple 8K SNP array v1 (www.illumina.com; [17]), based on the Infinium® II technique (Illumina Inc., Hayward, USA). Genomic DNA (gDNA) was extracted from each seedling using the NucleoSpin® Plant II kit (Macherey-Nagel GmbH and Co KG, Düren, Germany), and quantified using the Quant-iT™ PicoGreen® Assay (Invitrogen). Two-hundred nanograms of gDNA were used as template for the reaction, following the manufacturer's instructions. SNP genotypes were scored using the Genotyping Module (version 1.8.4) of the Illumina® GenomeStudio software (Illumina Inc.). The reliability of each genotype call was measured using the *GenCall* score set at a minimum of 0.15, which is a lower bound for calling genotypes relative to its associated cluster. SNPs were subsequently discarded using a sequence of criteria in the following order: *GenCall* score at the 50% rank (*50% GC*)<0.40; cluster separation (*ClusterSep*)<0.25; more than 5% missing calls; segregation discrepancy. The BEAGLE 3.1 software [43] was then used for imputing missing SNP genotypes.

### Estimation of linkage disequilibrium and genomic selection models

The degree of LD between SNPs was quantified with the parameter *r* ^2^ [44], estimated using GOLD software [45].

As the aim of our project was to predict BV, we developed models by fitting only the additive effects at each SNP. The two methods used for prediction of BV in this study were RR-BLUP and Bayesian LASSO ([3], [12], [46]. Both methods differ in terms of the prior assumptions about the distribution of the SNP effects, but the basic model is:
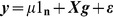
(2)where ***y*** is a vector of *n* BLUP-BVs for a given trait obtained from equation 1; μ is an intercept, **1_n_** is a vector of 1 s; ***X*** is a (*n×m*) design matrix allocating records to the *m* SNP effects, with element *X_ij_* = 0, 1, or 2 if the genotype of seedling *i* at SNP*_j_* is AA, AB, or BB, respectively; ***g*** is a (*m×*1) vector of SNP effects. For RR-BLUP, ***g*** is assumed to be normally distributed, *g_i_*∼*N*(0, 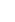); ***ε*** is a vector of random deviates with a variance of 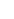. RR-BLUP was implemented in R 2.10.1 [47]. For the Bayesian LASSO method, ***g*** was assigned a prior distribution of double exponential (DE), 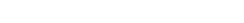. The DE distribution induces a strong shrinkage (very close to zero) of estimates for SNPs with relatively small effects and less shrinkage of estimates for SNPs with moderate or large effect; the residual variance (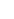) was assigned a scaled inverse chi-square prior distribution. The Bayesian LASSO method was implemented using the R/BLR package [46].

In both models, GEBV were estimated for the validation population as:
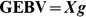
(3)where GEBV is the vector of breeding values estimated from the marker genotypes.

### Predicting unobserved BLUP-BV

The training population data was divided into two subsets: 90% of the training individuals were randomly selected for developing the prediction equation and the remaining 10% were used for cross validation. We repeated our analysis 10 times, and each time the prediction and validation sets of seedlings were randomly sampled and analyzed independently. The correlation coefficient between the observed (i.e., BLUP-BV) and the predicted (i.e., GEBV) breeding values was used as a measure of the accuracy of the GEBV prediction. The observed BLUP-BVs were linearly regressed on the predicted GEBV, where the regression coefficient reflected the degree of bias of the GEBV prediction and a regression coefficient of one indicates no bias.

As described in the preceding paragraph, the accuracy of GS in empirical studies is calculated as correlation between GEBV and BLUP-BV (i.e., *r*(GEBV, BLUP-BV)). Ideally one would also be interested in correlation between GEBV and true breeding values (TBV), i.e. *r*(GEBV,TBV), which could directly be used to compare the relative efficiency of GS with that of conventional BLUP-based selection as:
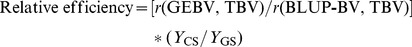
(4)


An estimate of *r*(GEBV,TBV) can be obtained as [*r*(GEBV, BLUP-BV)/*r*(BLUP-BV, TBV)] as suggested by [12]. The accuracy of conventional BLUP-based individuals selection (i.e., *r*(BLUP-BV, TBV)) in outbred full-sib families was calculated following [9]. The length of the conventional breeding cycle (*Y*_CS_) in New Zealand conditions is seven years, including three years for the assessment of phenotype. Since GS would obviate the need of phenotyping, the breeding cycle length for GS (*Y*_GS_) was assumed four years.
